# Effects of agitation on particle-size distribution and enzymatic hydrolysis of pretreated spruce and giant reed

**DOI:** 10.1186/1754-6834-7-77

**Published:** 2014-05-23

**Authors:** Adnan Kadić, Benny Palmqvist, Gunnar Lidén

**Affiliations:** 1 Department of Chemical Engineering, Lund University, Box 124, SE-221 00 Lund, Sweden

**Keywords:** Lignocellulose, Hydrolysis, Mixing, Particle-size distribution

## Abstract

**Background:**

Mixing is an energy demanding process which has been previously shown to affect enzymatic hydrolysis. Concentrated biomass slurries are associated with high and non-Newtonian viscosities and mixing in these systems is a complex task. Poor mixing can lead to mass and/or heat transfer problems as well as inhomogeneous enzyme distribution, both of which can cause possible yield reduction. Furthermore the stirring energy dissipation may impact the particle size which in turn may affect the enzymatic hydrolysis. The objective of the current work was to specifically quantify the effects of mixing on particle-size distribution (PSD) and relate this to changes in the enzymatic hydrolysis. Two rather different materials were investigated, namely pretreated Norway spruce and giant reed.

**Results:**

Changes in glucan hydrolysis and PSD were measured as a function of agitation during enzymatic hydrolysis at fiber loadings of 7 or 13% water-insoluble solids (WIS). Enzymatic conversion of pretreated spruce was strongly affected by agitation rates at the higher WIS content. However, at low WIS content the agitation had almost no effect on hydrolysis. There was some effect of agitation on the hydrolysis of giant reed at high WIS loading, but it was smaller than that for spruce, and there was no measurable effect at low WIS loading. In the case of spruce, intense agitation clearly affected the PSD and resulted in a reduced mean particle size, whereas for giant reed the decrease in particle size was mainly driven by enzymatic action. However, the rate of enzymatic hydrolysis was not increased after size reduction by agitation.

**Conclusions:**

The impact of agitation on the enzymatic hydrolysis clearly depends not only on feedstock but also on the solids loading. Agitation was found to affect the PSD differently for the examined pretreated materials spruce and giant reed. The fact that the reduced mean particle diameter could not explain the enhanced hydrolysis rates found for spruce at an elevated agitation suggests that mass transfer at sustained high viscosities plays an important role in determining the rate of enzymatic hydrolysis.

## Introduction

The biomass-based production of fuels and chemicals will be necessary in order to reduce both our oil dependency and our carbon footprint. Biorefineries utilizing the sugar platform to produce (at the moment mainly) fuels are on the verge of commercialization [1,2] and for these to succeed they will need to operate at high biomass loadings. A high solids loading is critical in order to produce sufficiently high concentrations of sugars during enzymatic hydrolysis and hence allow high final product titres. However, it is known that working at high biomass loadings introduces process difficulties, usually with decreasing hydrolysis yields as a result [3]. The ‘high solids effect’ has been widely discussed, but no fully satisfactory explanation has been found for the yield reduction. Possible explanations include incomplete homogenization/mixing of the material as a result of the high viscosity of biomass suspensions at elevated solids loading [4-8], increased end product inhibition as a consequence of higher cellobiose and glucose concentrations (possibly caused by large concentration gradients) [9], and/or the reduced availability of free water in the system [10].

At high solids loading the need for adequate mixing is vital, not only for the obvious reasons of avoiding concentration gradients and being able to accurately control pH and temperature, but also because mixing could provide synergistic effects with enzymatic hydrolysis (such as enhanced particle size reduction) as has been suggested [11]. Moreover, we have previously reported a strong positive effect of agitation on the enzymatic hydrolysis rate of spruce [12] and recently further showed that different types of materials behave differently in terms of influence of mixing during the hydrolysis [13].

Different mixing designs may be more suitable for different materials. Horizontal reactors with rotating paddles (tumbling mixers) have been shown to be quite effective for the mixing of fibrous pretreated materials like wheat straw and corn stover [14,15]. It is less clear if tumbling mixing would be as effective for the mixing of pretreated woody biomass, keeping in mind the large differences in initial viscosity and speed of liquefaction between the materials.

The relative importance of agitation in a stirred tank reactor may be in part determined by the characteristics of the pretreated material. We have previously reported that a much lower energy input is needed for the agitation of giant reed compared to spruce, possibly due to faster liquefaction during the enzymatic hydrolysis of giant reed [13]. The higher sustained specific power input during hydrolysis of spruce material could potentially cause physical damage to the material, with reduced particle sizes as a result. Reduced particle size has been correlated with increased hydrolysis rates and conversion levels in cellulose. Higher enzymatic hydrolysis rates have been observed in smaller particle sizes of red oak sawdust [16] and microcrystalline cotton cellulose [17]. Milling preceding hydrolysis has been shown to increase hydrolysis rates and conversion levels in microcrystalline cotton cellulose [17], while no effects of milling on the hydrolysis of dilute acid-pretreated corn stover were observed [18]. Particle size reduction caused by the physical effect of agitation, similar to milling, may be occurring simultaneously with enzymatic hydrolysis in a stirred tank reactor. Since decreased particle size is associated with increased specific external surface area, an increased adsorption of cellulases could possibly explain the positive effect of agitation on the hydrolysis rate.

The objective of the current study was to characterize the particle-size distribution (PSD) during enzymatic hydrolysis of two different types of pretreated biomass - Norway spruce and giant reed - at various levels of agitation. Furthermore, changes in PSD measured during hydrolysis were compared to PSD changes caused by pure agitation in the absence of hydrolysis in order to decouple the specific mechanical effect on PSD from the reduction due to hydrolysis.

## Results and discussion

Three different impeller speeds (100, 300 and 600 rpm) were chosen to represent different levels of specific power input during hydrolysis. It was hypothesized that the high specific power input at high impeller speed would cause physical damage to the spruce material, which could be observed by measuring PSD. Enzymatic hydrolysis was performed at two different solids loadings, 7% and 13% water-insoluble solids (WIS). This was done in order to eliminate stagnant zones even at low impeller speeds, which could be achieved at 7% WIS content due to the low viscosity of the diluted material, and thus isolate the effects of power input and mixing.

### Viscosity of the pretreated material

**Figure 1:**
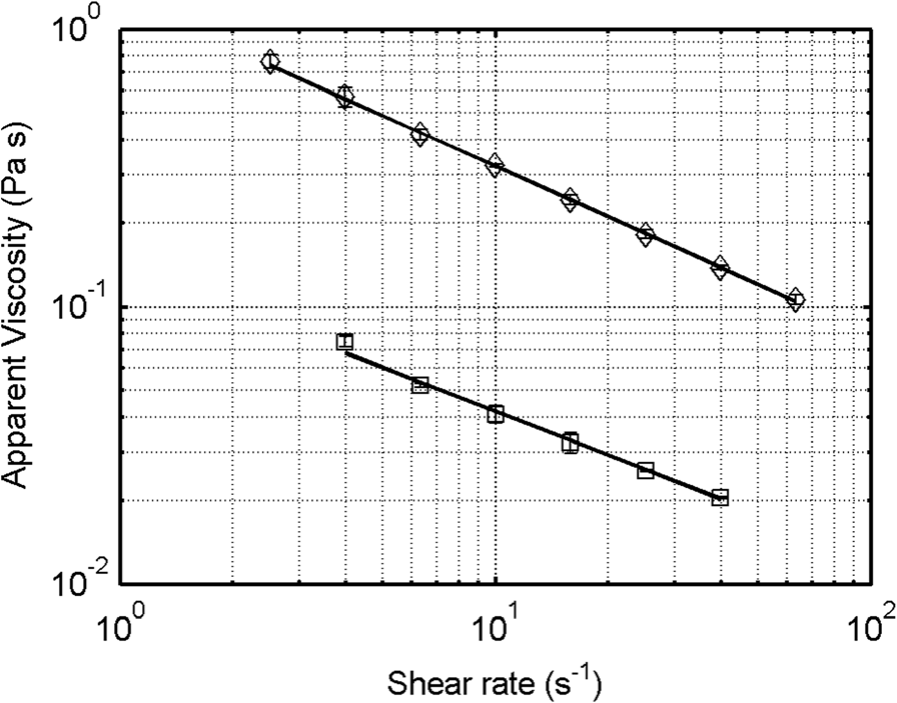
**Flow curves for spruce at different WIS loading.** Markers: Square represents spruce at 7% WIS and diamond represents spruce at 13% WIS. The lines represent fits of the power-law viscosity model to the data sets. WIS, water-insoluble solids.

The viscosity of pretreated spruce at 7 and 13% WIS content was measured in a rotational rheometer. It was not possible to perform corresponding measurements with giant reed due to the solid-like properties of the material. The flow curves of pretreated spruce exhibited non-Newtonian, shear-thinning behavior (Figure 1), as previously reported for a similar material [8]. Estimated power-law parameters for spruce at 7 and 13% WIS are shown in Table 1. Spruce at 13% WIS loading exhibited very high apparent viscosity, greater than 0.1 Pa s, over a wide range of shear rates. The apparent viscosity was highly dependent on WIS loading, being an order of magnitude higher at 13% WIS than at 7% WIS. The similar influence of solids loading on viscosity has been reported in spruce [8] and corn stover slurries [7].

**Table 1:** Fitted values of the parameters K and n in the power-law viscosity model of spruce slurry at 7 and 13% water-insoluble solids

**WIS**	***K***	***n***	**R**^**2**^
7%	0.14	0.47	0.995
13%	1.30	0.39	0.999

### The effect of agitation on enzymatic hydrolysis

**Figure 2:**
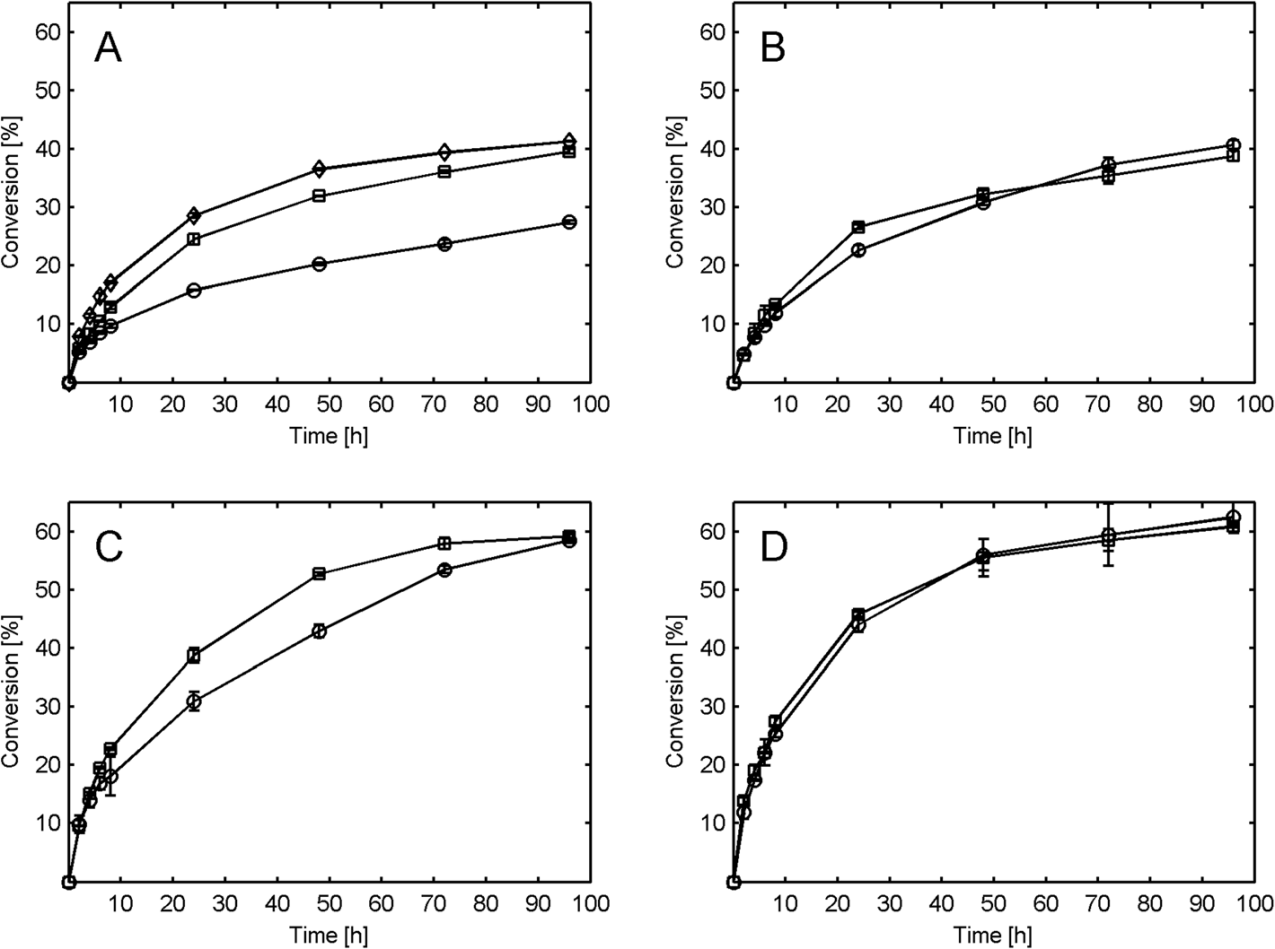
**Conversion of glucan to glucose during enzymatic hydrolysis at different impeller speeds. (A)** Spruce at 13% WIS. **(B)** Spruce at 7% WIS. **(C)** Giant reed at 13% WIS. **(D)** Giant reed at 7% WIS. Markers: Diamond represents 600 rpm, square represents 300 rpm and circle represents 100 rpm. h, hours; WIS, water-insoluble solids.

Enzymatic hydrolysis experiments at varying agitation rates were conducted for both Norway spruce and giant reed material at two different WIS loadings (7 and 13%). The conversion of glucan to glucose in spruce at 13% WIS loading was strongly affected by agitation, with conversions after 48 hours reaching 20, 32 and 37% for 100, 300 and 600 rpm respectively (Figure 2A). Large stagnant zones were observed throughout the hydrolysis at 100 rpm which could explain the decrease in yield if mass transfer limitations were present at lower agitation rates. These results are consistent with what has previously been reported on pretreated spruce [12].

On the other hand, the conversion of glucan to glucose in giant reed at 13% WIS loading was less affected by agitation, with conversions after 48 hours reaching 43% for 100 rpm and 53% for 300 rpm (Figure 2C). However, the effect was only temporary and the same final conversion level was reached after 96 hours for the two stirrer rates. Large stagnant zones were initially present at both impeller speeds, but - judged by visual inspection - the liquefaction proceeded faster at 300 rpm, possibly due to better mixing.

When decreasing the WIS content to 7%, and hence lowering the viscosity of the slurries, the conversion of glucan to glucose for both spruce and giant reed was generally unaffected by agitation. The conversion of spruce after 48 hours was 31% for 100 rpm and 32% for 300 rpm and the conversion of giant reed after 48 hours was 56% for 100 rpm and 56% for 300 rpm (Figure 2B and D). The low viscosity at 7% WIS allowed the impeller to provide motion throughout the reactor volume, even at low impeller speed. This may have removed mass transfer limitations that could appear in stagnant material.

The effects of agitation may be linked to material properties since spruce at high WIS loading is a highly viscous material that, as previously reported [13], maintains much of its viscosity during hydrolysis, for example only a five-fold decrease in viscosity was observed during 48 hours of hydrolysis in a similar material [8]. In contrast, liquefaction of pretreated grasses proceeds quickly, for example more than a 10-fold reduction in viscosity occurred during the first 8 hours of corn stover hydrolysis at comparable WIS loading [15] and a similar rapid decrease in viscosity was observed during the first two to six hours of giant reed hydrolysis in an anchor impeller reactor [13].

This may explain the effect of agitation on giant reed seen here. The pitch-blade impeller used was not able to provide sufficient initial mixing at low impeller speed. The result was slower liquefaction at 100 rpm and stagnant zones that were maintained for a longer time period. This possibly led to slower hydrolysis until liquefaction was achieved.

### Changes in particle-size distribution during hydrolysis

The elevated viscosity of spruce at high WIS loading leads to higher specific power inputs for mixing and it has been shown that hydrolysis may even proceed faster at higher WIS loading at the same impeller speed [13]. This observation indicates that agitation potentially causes damage to the fibers in spruce which could make the cellulose more accessible or easier to hydrolyze. One way to investigate this is to measure changes in PSD at different impeller speeds.

**Figure 3:**
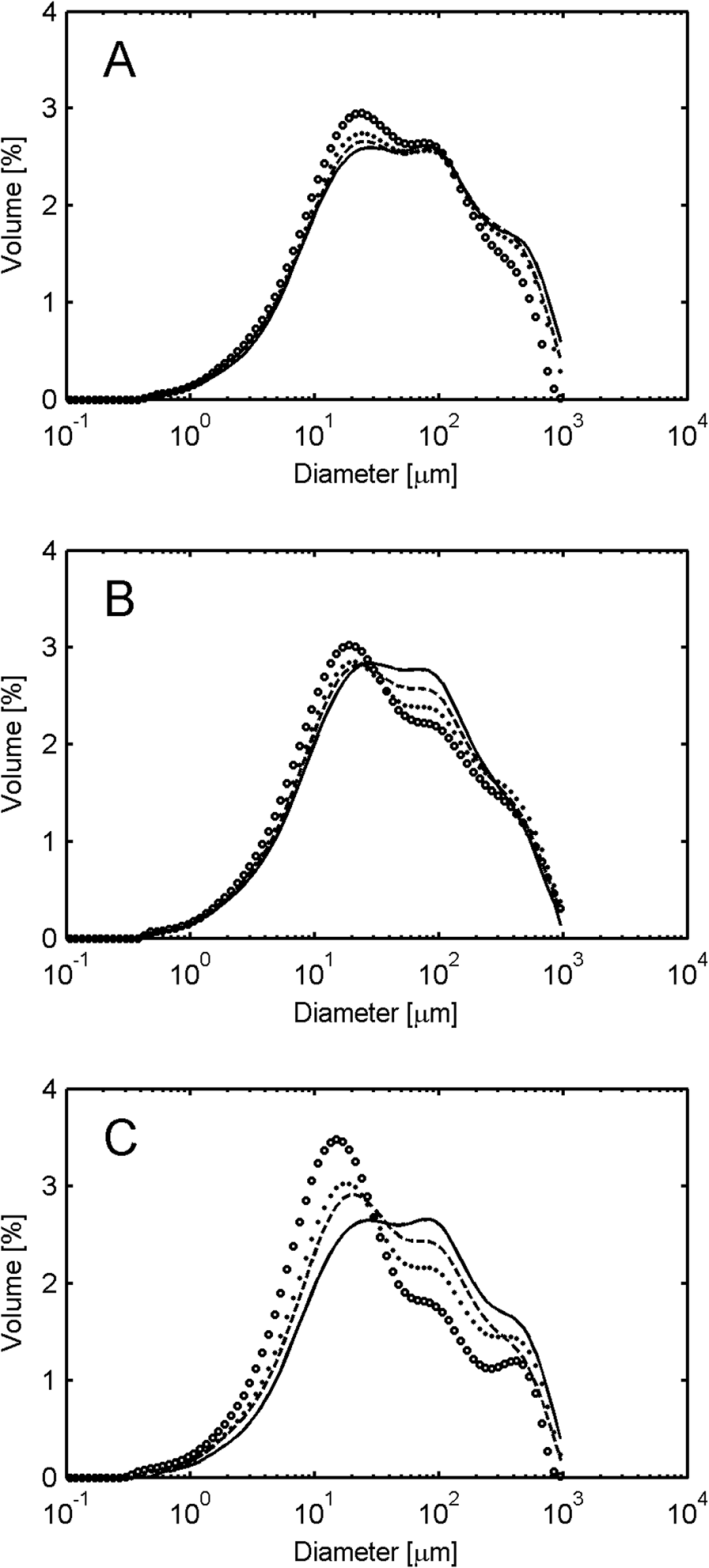
**Volume-based particle-size distribution during enzymatic hydrolysis of spruce at 13% water-insoluble solids. (A)** Impeller speed of 100 rpm. **(B)** Impeller speed of 300 rpm. **(C)** Impeller speed of 600 rpm. Markers: one dash line represents 0 hours, two dash line represents 4 hours, filled circles represents 24 hours, and empty circles represents 96 hours.

In this study, the measured volume-based PSD showed almost no change during the hydrolysis of 13% WIS spruce at 100 rpm (Figure 3A), and even though glucan degradation was observed, it seems the enzymes were not able to separate the fibers into smaller particles to any larger extent.

As the impeller speed was increased, a higher rate of particle size reduction was observed (Figure 3B and C). The change was associated with the enrichment of particles in a 10 to 20 μm size peak coupled with the decreasing contribution of larger size particles to the total volume. For a version of Figure 3 with estimated standard deviations, see Additional file 1.

This reduction in particle size indicates that physical forces exerted on the spruce particles by agitation caused fiber separation and damage to the fibers. The peak in the distribution at 10 to 20 μm may be related to the fiber thickness in pretreated spruce. Fiber diameters in the range of 20 to 40 μm have been reported for Norway spruce [19]. The decrease in particle size may make the cellulose more hydrolysable due to an increased specific surface area. Higher hydrolysis rates have indeed been reported for smaller particle sizes of untreated red oak sawdust [16]. However, in the mentioned study, it was not clear if other characteristics of the material were different in the various particle sizes examined. Additionally, the large particle sizes had higher viscosity which may have caused differing flow conditions during hydrolysis. In contrast, fiber length was shown to have no effect on hydrolysis rates in organosolv-pretreated softwood, the hydrolysis rate instead being correlated to cellulose crystallinity and accessibility [20]. Similarly, the fiber length had no effect on the hydrolysis rate of poplar fibers derived from the sulphate and sulphite processes [21]. Thus, it is unclear if a smaller particle size per se is correlated to a more hydrolysable material.

**Figure 4:**
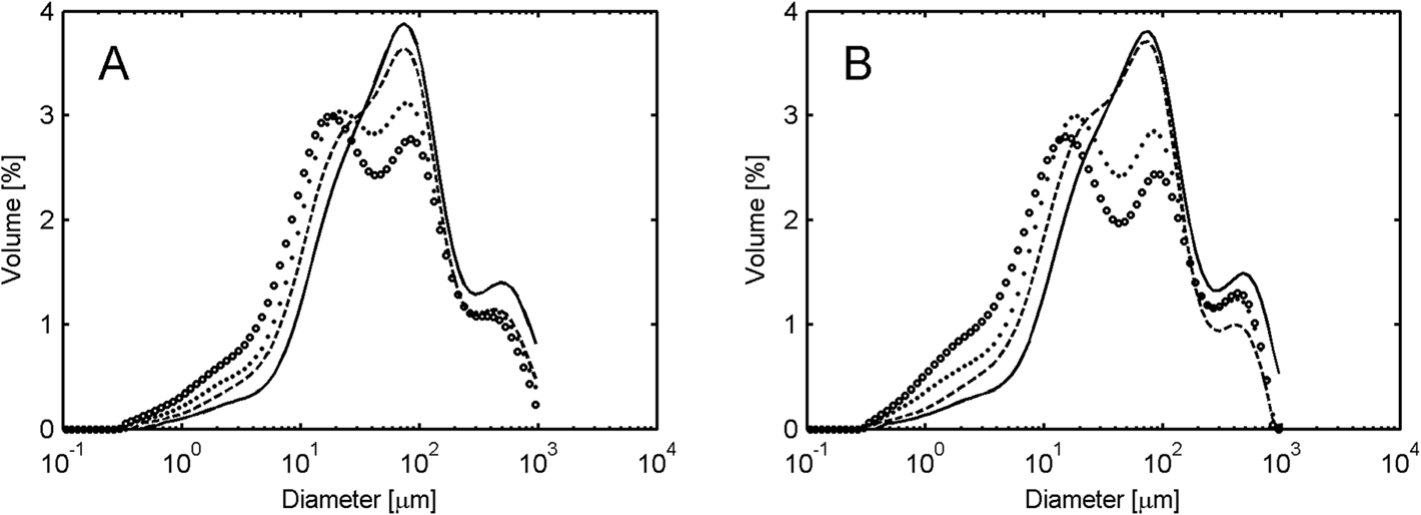
**Volume-based particle-size distribution during enzymatic hydrolysis of giant reed at 13% water-insoluble solids. (A)** Impeller speed of 100 rpm. **(B)** Impeller speed of 300 rpm. Markers: one dash line represents 0 hours, two dash line represents 4 hours, filled circles represents 24 hours, and empty circles represents 96 hours.

The initial volume-based PSD of giant reed showed a greater fraction of large particles and a larger maximum particle size compared to spruce, which was in accordance with visual observation. During enzymatic hydrolysis, a fast reduction in size was observed at both high and low impeller speed, and the particles were reduced to smaller sizes than in spruce. However, an enrichment of particles in a 10 to 20 μm size peak was again observed (Figure 4A and B). For a version of Figure 4 with estimated standard deviations, see Additional file 2.

For hydrothermally pretreated wheat straw it has been shown that endoglucanases selectively bind to dislocations which regularly appear across the fibers. The hydrolysis of the material leads to the shortening of the fibers to a length corresponding to the minimum distance between the dislocations, that distance being equal to the 20 μm diameter of the fibers [22]. These results correspond well with the 10 to 20 μm peak size observed here. Surprisingly, at 7% WIS both giant reed and spruce exhibited similar changes in PSD as the respective material at 13% (data not shown), although no effect on the hydrolysis rate was observed (Figure 2B and D).

**Figure 5:**
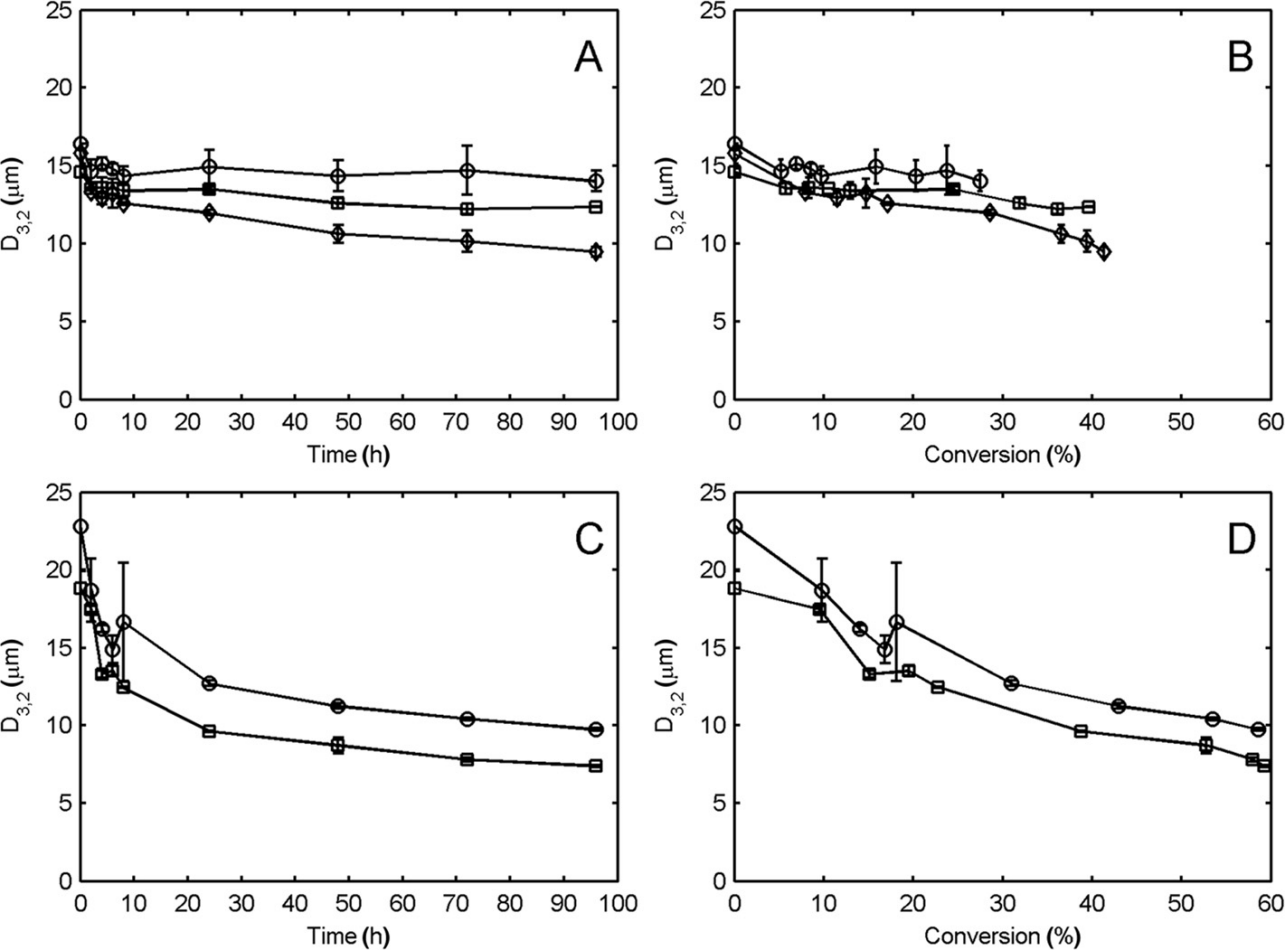
**Surface-weighted mean diameter (D**_**3,2**_**) during enzymatic hydrolysis at different impeller speeds. (A, B)** Spruce at 13% WIS. **(C, D)** Giant reed at 13% WIS. Markers: Diamond represents 600 rpm, square represents 300 rpm, and circle represents 100 rpm. WIS, water-insoluble solids.

By integration of the PSD, a mean particle diameter can be estimated. The resulting surface-weighted mean diameter (D_3,2_) of spruce at 13% WIS decreased faster at higher impeller speeds (Figure 5A). Interestingly, when the average diameters were plotted against the corresponding conversion levels instead of time, it was obvious that higher impeller speeds caused smaller particle size even at the same extent of hydrolysis (Figure 5B). This indicates that agitation caused particle size reduction due to physical forces exerted on the particles.

The reduction of D_3,2_ during giant reed hydrolysis was faster than in spruce and relatively less dependent on agitation intensity (Figure 5C). Moreover, the decrease in size was not necessarily coupled to the higher hydrolysis rate, as the main size reduction in giant reed was seen during the initial hours when only a small part of the material had been hydrolyzed. Furthermore, the size reduction in giant reed was substantially larger even in the same conversion interval as spruce (Figure 5D). The quick reduction in particle size may be a part of the explanation for the much faster liquefaction of giant reed compared to spruce.

**Figure 6:**
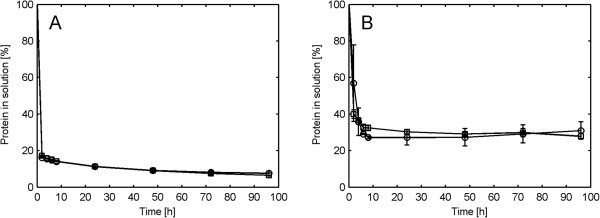
**Protein adsorption during enzymatic hydrolysis at different impeller speeds. (A)** Spruce at 13% WIS. **(B)** Giant reed at 13% WIS. Markers: Square represents 300 rpm and circle represents 100 rpm. WIS, water-insoluble solids.

Since an increased agitation rate was correlated with a smaller particle size, a larger total surface area could also be expected. This would potentially increase the enzyme adsorption and hence also explain the increased hydrolysis rates seen under more intense agitation. However, the results showed that protein adsorption during spruce hydrolysis at 13% WIS was independent of the impeller speed (Figure 6A). Most of the protein in the enzyme cocktail was adsorbed at the start of hydrolysis regardless of agitation rate (83% adsorbed after two hours of hydrolysis at 300 rpm). This indicates that the higher conversion levels achieved at higher impeller speeds were not caused by an increased adsorption of cellulases. It is possible that the total available surface area (internal plus external) in the pretreated material was already sufficiently large, and therefore additional area created by physical disruption of the particles did not lead to increased enzyme adsorption.

The adsorption results are well in accordance with previously reported values where more than 95% adsorption of endoglucanases and cellobiohydrolases on steam pretreated spruce was observed [23]. The protein adsorption during giant reed hydrolysis at 13% WIS was also found to be independent of the impeller speed (Figure 6B) and the most notable difference between the materials was less adsorbed protein during giant reed hydrolysis compared to spruce hydrolysis.

### The effect of agitation on particle-size distribution and its effect on the rate of hydrolysis

**Figure 7:**
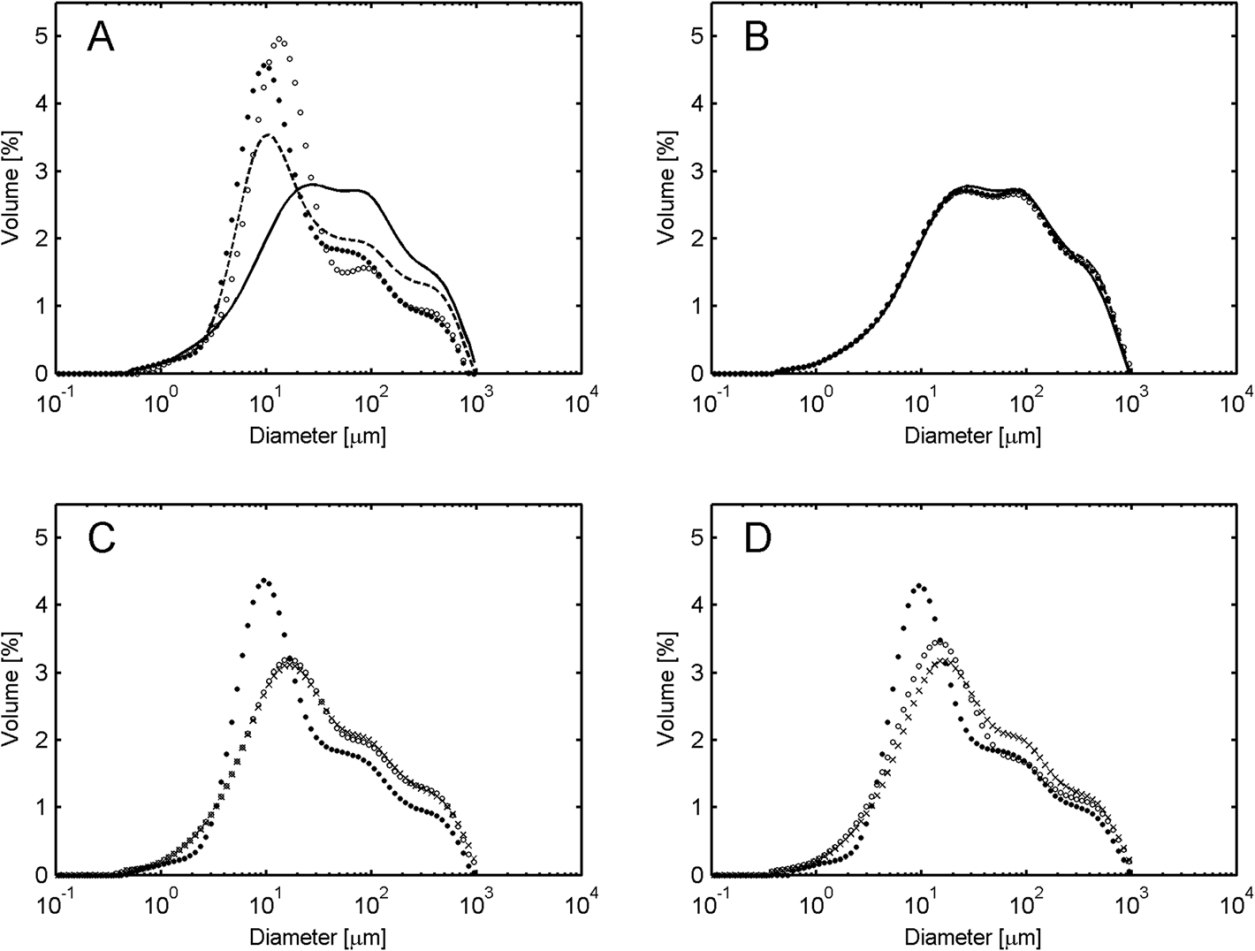
**Volume-based particle-size distribution during agitation and subsequent enzymatic hydrolysis.** Material used: spruce at 13% WIS. **(A)** Agitation for 48 hours at 600 rpm followed by 48 hours of agitation at 100 rpm. **(B)** Agitation for 96 hours at 100 rpm. **(C)** Agitation for 48 hours at 600 rpm followed by hydrolysis for 48 hours at 100 rpm (only hydrolysis shown). **(D)** Agitation for 48 hours at 600 rpm followed by hydrolysis for 48 hours at 600 rpm (only hydrolysis shown). Markers: dash line represents 0 hours, two dash line represents 24 hours, filled circles represents 48 hours, x represents 50 hours, and empty circles represents 96 hours.

In order to investigate if particle size reduction was caused by the physical effects of agitation rather than enzymatic action, spruce material at 13% WIS was agitated at 600 rpm without enzyme addition. A fast decrease in particle size was observed during the first 48 hours of pure agitation at 600 rpm (Figure 7A) and a strong enrichment of particles in the size range 10 to 20 μm was observed. This was clear evidence for a physical effect of agitation on particle size reduction. When the impeller speed was decreased to 100 rpm for the following 48 hours the particle size remained relatively constant, indicating irreversible physical damage to the spruce particles (Figure 7A). A negative control was performed by agitating 13% WIS spruce at 100 rpm without enzyme addition, with no change in PSD being observed (Figure 7B).

Because of the solid-like properties of the giant reed material, it was impossible to mix it in the reactor without enzyme addition, thus corresponding experiments could not be performed on giant reed.

In order to investigate if the change in particle size had an effect on the hydrolysis rate, spruce at 13% WIS was agitated for 48 hours at 600 rpm without enzyme addition. After this initial step, which reduced the particle size, enzymes were added. The subsequent hydrolysis was performed at both 100 and 600 rpm for 48 hours. The 10 to 20 μm size peak formed before enzyme addition disappeared during the first two hours of hydrolysis and after that the PSD remained constant during hydrolysis at 100 rpm, while it started to shift to smaller sizes again at 600 rpm (Figure 7C and D).

**Figure 8:**
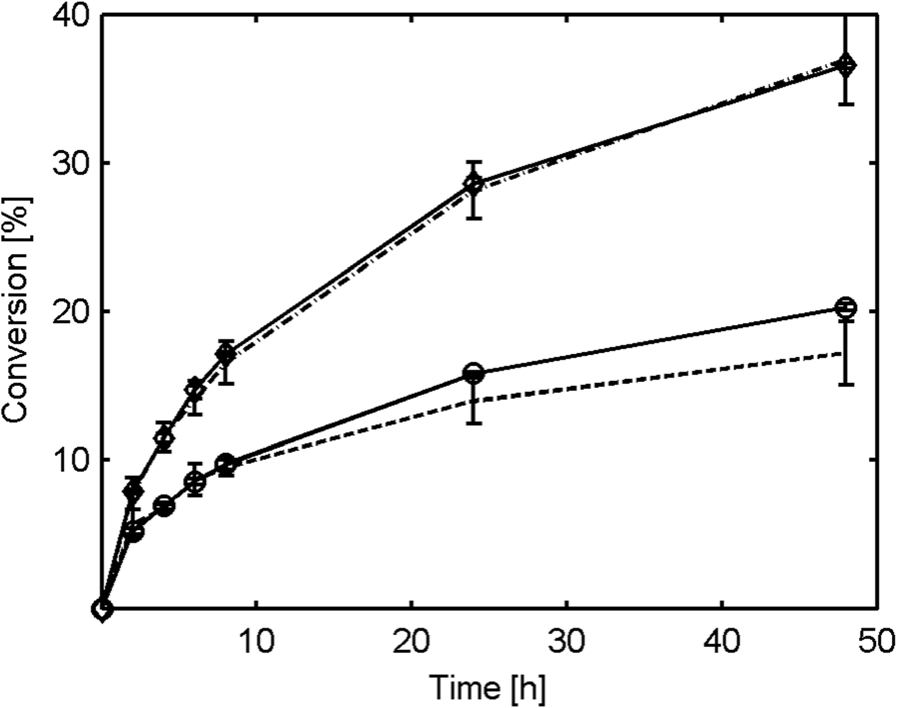
**Conversion of glucan to glucose during enzymatic hydrolysis after 48 hours of agitation.** Material used: spruce at 13% water-insoluble solids. Markers: Diamond represents hydrolysis at 600 rpm, empty circle represents hydrolysis at 100 rpm, two dash line represents hydrolysis at 100 rpm after 48 hours of agitation at 600 rpm, and dash line represents hydrolysis at 600 rpm after 48 hours of agitation at 600 rpm.

Interestingly, the decrease in particle size caused by 48 hours of agitation without added enzymes had little effect on the conversion levels achieved in the subsequent 48 hours of hydrolysis (Figure 8) and the rate of conversion was mostly determined by the agitation rate during the hydrolysis. This strongly indicates that the enhanced hydrolysis rates seen at increased agitation levels cannot be explained merely by the physical damage to the particles induced by intensive agitation.

## Conclusions

In this study it was found that an increased agitation rate caused large changes in PSD during spruce hydrolysis. However, the agitation rate only had an effect on the enzymatic hydrolysis of spruce at high WIS loading (13%), and not at a lower WIS loading (7%). Furthermore, the hydrolysis rate of giant reed was only slightly affected at high WIS loading (13%) and not at all at low WIS loading (7%). It was moreover shown that higher agitation rates, rather than the enzymatic action, caused particle size reduction in spruce and that the change in hydrolysis rate could not be coupled with the difference in PSD. In contrast, particle size reduction in giant reed was mostly driven by enzymatic hydrolysis, with a fast initial decrease in particle size that could potentially explain why liquefaction of this material is achieved after only a short period of time, while being largely absent in spruce. These results support the idea that high viscosity, which is retained over time, causes mass transfer limitations that diminish the rate of enzymatic hydrolysis.

## Methods

### Raw material and pretreatment

Two different materials were used in this study. Norway spruce (*Picea abies*) pretreated by SO_2_ catalyzed steam pretreatment was kindly provided by Sekab (Örnsköldsvik, Sweden). The material was stored at 4°C until used. Giant reed (*Arundo donax*), pretreated without added chemicals, was provided through the BIOLYFE project and stored frozen until used.

The compositions of the giant reed solid fraction, and the solid and liquid fraction of the pretreated spruce (Table 2), were analyzed using the National Renewable Energy Laboratory (NREL) standard procedure [24,25]. The WIS content of the pretreated materials was measured by washing the materials repeatedly with deionized water over filter paper (number 1; Whatman, Florham Park, New Jersey, United States). The WIS content of giant reed and spruce were determined to be 28% and 17% (wt/wt) respectively.

**Table 2:** Composition of solid and liquid fractions of the pretreated materials

**Spruce solids**	**% of WIS**	**Spruce liquid**	**g/L**	**Giant reed solids**	**% of WIS**
Glucan	53.8	Glucose	32.4	Glucan	53.8
Mannan	nd	Mannose	40.0	Mannan	nd
Galactan	nd	Xylose	15.5	Galactan	nd
Xylan	nd			Xylan	9.6
Arabinan	nd			Arabinan	nd
Lignin	43.3			Lignin	31.3

The WIS content of giant reed and spruce were determined to be 28% and 17% (wt/wt) respectively. (nd, not detected, below the sensitivity of the instrument). WIS, water-insoluble solids.

### Rheological analysis of pretreated material

The flow curve measurements were carried out on whole slurry samples using a rotational rheometer (Kinexus, Malvern Instruments, Malvern, United Kingdom) set at 45°C. Measurements were performed only on spruce slurry at 7 and 13% WIS, it was not possible to attain corresponding flow curves for giant reed because of the solid-like properties of the material. In order to avoid slip at the rotating part, a four-bladed vane (diameter: 21 mm, length: 61 mm, vane width: 1.5 mm) was employed [26-28]. A splined cup (diameter: 27.5 mm, cup wall height: 62.5 mm) was used as the stationary part. Flow curve measurements were carried out using shear-rate control in the range of 1 to 100 per second. Measurements were performed in five steps per decade with logarithmic spacing. Every step included 15 seconds of shearing at the new shear rate, in order to ensure a steady shearing, followed by measuring for 10 seconds at the same shear rate. The reported data are integrated mean values from these 10 seconds. Data points sampled for non-laminar flows, indicated by a sudden increase in shear stress at higher shear rates, are not presented. All measurements were carried out in duplicates. A simple power-law model of viscosity (Equation 1) was fitted to the data by using the non-linear regression function *nlinfit* in Matlab 7.13 (Mathworks, Natick, Massachusetts, United States):

(1) $$\tau =K\cdot {\dot{\gamma }}^{n}$$

where *τ* represents the shear stress (Pa), $\overset{˙}{\gamma }$ represents the shear rate (s^−1^) and K and n are the fitted model parameters. Apparent viscosity was defined as the quotient of shear stress and shear rate.

### Hydrolysis experiments

All hydrolysis experiments were carried out in 2.5 L bioreactors: Biostat A (B. Braun Biotech International, Melsungen, Germany) and Biostat A Plus (Sartorius, Melsungen, Germany), with a diameter (D_t_) of 130 mm. The reactors were equipped with a pitched-blade impeller with three blades at an angle of 45°, a diameter (D_i_) of 70 mm and a blade width (w_i_) of 20 mm, pumping upwards. The working weight was 1.0 kg.

The WIS content of the pretreated materials was adjusted to 7% or 13%. In all cases, with the exception of spruce at 7% WIS, sterile deionized water was used as the diluent. Sterile deionized water combined with the liquid fraction of pretreated spruce was used to dilute the spruce material to 7% WIS. This was done in order to ensure the same initial concentration of sugars and inhibitors in the liquid at 7% and 13% WIS. In all cases the slurry was adjusted to a pH of 5.0 prior to the start of the hydrolysis experiments by the addition of 12 M NaOH.

All hydrolysis experiments were performed at 45°C. In order to ensure an even temperature distribution at low impeller speeds the reactors were placed in temperature-controlled water baths. The enzyme preparation used (Cellic CTec2) was kindly provided by Novozymes A/S (Bagsvaerd, Denmark). All experiments were conducted at an enzyme loading of 0.05 g enzyme solution/g WIS, corresponding to 4.5 FPU (Filter Paper Unit)/g WIS.

Experiments using spruce were initiated by 10 seconds of intense mixing at 600 rpm to ensure an even initial distribution of the enzymes. The same was achieved for giant reed by mixing in the enzymes with a spoon. Every subsequent sampling was preceded by 10 seconds of intense mixing at 600 rpm to assure a representative sample. Samples for sugar concentration, protein adsorption, and PSD measurements were taken throughout the hydrolysis. All hydrolysis experiments were performed in duplicate.

### Analysis of hydrolysis samples

Sugar concentrations were measured using high-performance liquid chromatography (HPLC). Samples from the hydrolysis liquid were separated in a centrifuge (Z 160 M, Hermle Labortechnik, Wehingen, Germany) in 2 mL eppendorf tubes at 13,000 rpm for 5 minutes. The supernatant was filtered through 0.2 μm filters and stored at -20°C. The sugar concentrations were determined using a polymer column (Aminex HPX-87P, Bio-Rad Laboratories, München, Germany) at 85°C. Deionized water (Purelab Flex, Elga, Marlow, United Kingdom) was used as an eluent at a flow rate of 0.6 mL/min. The sugars were detected with a refractive index detector (Waters 2410, Waters, Milford, Massachusetts, United States). The conversion of glucan to glucose was calculated using Equation 2:

(2) $$C_{g}=\frac{\left(c_{g}-c_{\mathrm{go}}\right)\cdot \frac{m}{\rho_{L}}\cdot \left(1-\mathrm{WIS}\right)}{m\cdot \mathrm{WIS}\cdot \varphi_{g}\cdot 1.11}$$

where *c*_*g*_ is the glucose concentration (g/L), *c*_*g*0_ is the initial glucose concentration (g/L), *m* is the working weight of the reactor (g), *ρ*_*L*_ is the initial liquid density, taken as the density of water (g/L), *WIS* is the initial mass fraction of water-insoluble solids and *φ*_*g*_ is the initial mass fraction of glucan in the WIS. The concentration of cellobiose during the hydrolysis experiments never exceeded 3 g/L.

Protein concentrations were measured using a Bradford assay [29]. Samples were prepared in the same manner as the samples for HPLC analysis but were stored on ice for no longer than 96 hours. The protein concentrations were measured with a commercial kit (Coomassie Plus (Bradford) Assay Kit, Thermo Fisher Scientific, Waltham, Massachusetts, United States). The standard curve was fitted as a second degree polynomial to bovine albumin standard in the range of 25 to 500 mg protein/L. Protein concentrations at the start of hydrolysis were calculated based on enzyme loading.

PSD was measured using laser diffraction analysis. Small samples (approximately 0.5 mL) were taken throughout the hydrolysis and kept on ice for no longer than four hours. The samples were transferred in their entirety to a sample dispersion unit (Hydro 2000SM, Malvern Instruments, Malvern, United Kingdom) and diluted with deionized water. The measurements were performed using a particle size analyzer (Mastersizer 2000, Malvern Instruments, Malvern, United Kingdom) connected to the sample dispersion unit. The PSD of each sample was based on an average of three 15 second measurements. The laser diffraction was analyzed using Mie’s theory. The refractive index and absorption coefficient for the lignocellulosic materials were taken as 1.5 and 1.0 respectively. These values were deemed satisfactory as the residual of most measurements was smaller than 0.3%. No measurement had a residual exceeding 1%. The results were extracted in the form of volume-based PSD and surface-weighted mean diameter D_3,2_.

## Competing interests

The authors declare that they have no competing interests.

## Authors’ contributions

AK participated in the design of the study, performed the experimental work, and wrote the manuscript. BP participated in the design of the study, performed the reactor hydrolysis experiments, and wrote the manuscript. GL participated in the design of the study and commented on the manuscript. All authors contributed to the scientific discussion throughout the work, and have read and approved the final manuscript.

## Supplementary Material

Additional file 1 **Volume-based particle-size distribution during enzymatic hydrolysis of spruce at 13% WIS. (A)** Impeller speed of 100 rpm. **(B)** Impeller speed of 300 rpm. **(C)** Impeller speed of 600 rpm. Markers: blue line 0 h, red line 4 h, green line 24 h, yellow line 96 h. Click here for file

Additional file 2 **Volume-based particle-size distribution during enzymatic hydrolysis of giant reed at 13% WIS.(A)** Impeller speed of 100 rpm. **(B)** Impeller speed of 300 rpm. Markers: blue line 0 h, red line 4 h, green line 24 h, yellow line 96 h. Click here for file
