# Supplementary material for: Effects of agitation on particle-size distribution and enzymatic hydrolysis of pretreated spruce and giant reed
Source: Biotechnol Biofuels. 2014 May 23;7:77. doi: 10.1186/1754-6834-7-77 (PMC4035727; doi:10.1186/1754-6834-7-77)
Supplement: Additional file 2 — Volume-based particle-size distribution during enzymatic hydrolysis of giant reed at 13% WIS.(A) Impeller speed of 100 rpm. (B) Impeller speed of 300 rpm. Markers: blue line 0 h, red line 4 h, green line 24 h, yellow line 96 h. [file 1754-6834-7-77-S2.pdf]

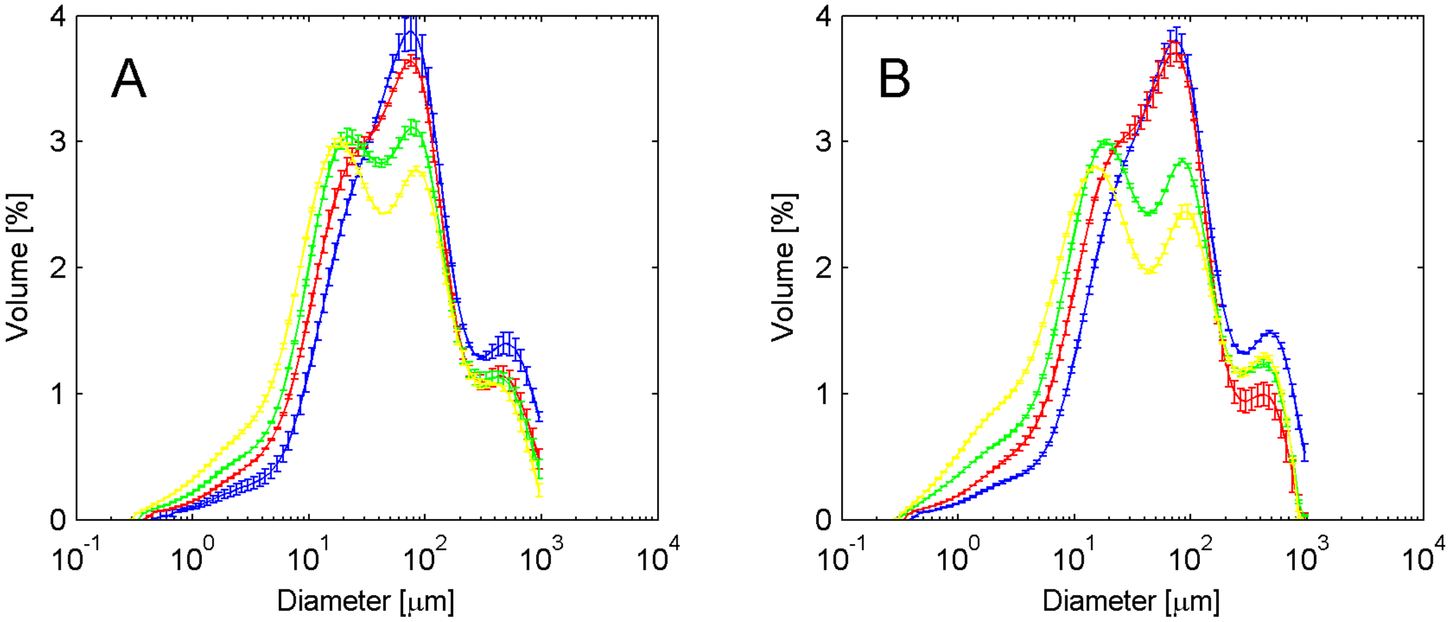


**Volume-based particle-size distribution during enzymatic hydrolysis of giant reed at 13% WIS. (A**) Impeller speed of 100 rpm. **(B)** Impeller speed of 300 rpm. **Markers:** blue line 0h, red line 4h, green line 24h, yellow line 96h.
